# Evaluating a CTSA-funded pilot grant program

**DOI:** 10.1017/cts.2020.557

**Published:** 2020-11-16

**Authors:** Kalene Morozumi, Tanha Patel, Paul Kerr, Mary Beth Cassely, Timothy Carey, John Buse, Andrea Carnegie, Tom Egan, Gaurav Dave

**Affiliations:** 1 North Carolina Translational and Clinical Sciences Institute, University of North Carolina at Chapel Hill, Chapel Hill, NC, USA; 2 Department of Medicine, University of North Carolina at Chapel Hill, Chapel Hill, NC, USA; 3 Department of Social Medicine, University of North Carolina at Chapel Hill, Chapel Hill, NC, USA; 4 Department of Surgery, University of North Carolina at Chapel Hill, Chapel Hill, NC, USA

**Keywords:** CTSA, evaluation, pilot funding, pilot program, translational research

## Abstract

**Introduction:**

Pilot programs are integral to catalyzing and accelerating research at Clinical and Translational Science Award (CTSA) hubs. However, little has been published about the structure and operationalization of pilot programs or how they impact the translational research enterprise at CTSAs. The North Carolina Translational and Clinical Science Institute (NC TraCS), the CTSA hub at the University of North Carolina at Chapel Hill (UNC-CH) conducted an evaluation case study to describe the pilot program structure, assess process outcomes, and provide a framework for other institutions to utilize for the evaluation of their respective pilot programs.

**Methods:**

We describe the operationalization of our pilot program, the evaluation framework utilized to evaluate the program, and how we analyzed available data to understand how our pilot funding opportunities were utilized by investigators. We calculated application volumes and funding rates by investigator position title and pilot application type. We also reviewed feedback provided by pilot Principal Investigators (PIs) to understand how many pilot projects were completed, NC TraCS service utilization, and barriers to research. Limited data on publications and subsequent funding was also reviewed.

**Results:**

Between 2009 and 2019 the NC TraCS Pilot Program received 2343 applications and funded 933 projects, ranging from $2000 to $100,000 in amount, with an overall funding rate of 39.8%. Utilization of NC TraCS services had positive impacts on both resubmission funding and project completion rates.

**Conclusion:**

This process evaluation indicates that the program is being operationalized in a way that successfully fulfills the program mission while meeting the needs of a diverse group of researchers.

## Introduction

Evidence suggests that interventions and innovations building on previous research have timelines that are on average 3 years shorter, which potentially translates to $100–200 million dollars in development costs [1]. While the efficiency of translating research from bench to bedside has improved, gaps in research discovery and innovation still continue to persist [1–3]. In particular, gaps in how research is translated from basic science to clinical studies have been suggested as limiting the pace of translational science [1–3]. High research costs, slow dissemination of findings, insufficient translational research workforce, and limited funding, especially for exploratory research, are some of the major barriers to translational research [1–3].

To address these gaps, the National Institutes of Health (NIH) established the Clinical and Translational Science Awards (CTSA) Program. The CTSA Program is a network of academic medical research institutions (called CTSA hubs) that work individually and collaboratively to improve the translational research process. Funded since 2008, the CTSA Program is tasked with developing innovative solutions to improve the process for translating clinical and laboratory research into interventions that will benefit the individual and public health [4].

The University of North Carolina-Chapel Hill (UNC-CH) CTSA award (first awarded in 2008) is administered by the North Carolina Translational and Clinical Sciences Institute (NC TraCS). NC TraCS has partnered with RTI International (RTI), North Carolina Agricultural and Technical State University (NC A&T) since 2013, and North Carolina State University (NCSU) since 2018 to improve the health of North Carolinians by: (1) providing access to research expertise; (2) developing and disseminating innovative tools, methods, and resources to facilitate translational research; and (3) building a skilled translational research workforce. One of the major activities of all CTSA hubs is administering pilot grants to encourage and facilitate novel clinical and translational research.

**Fig. 1.**
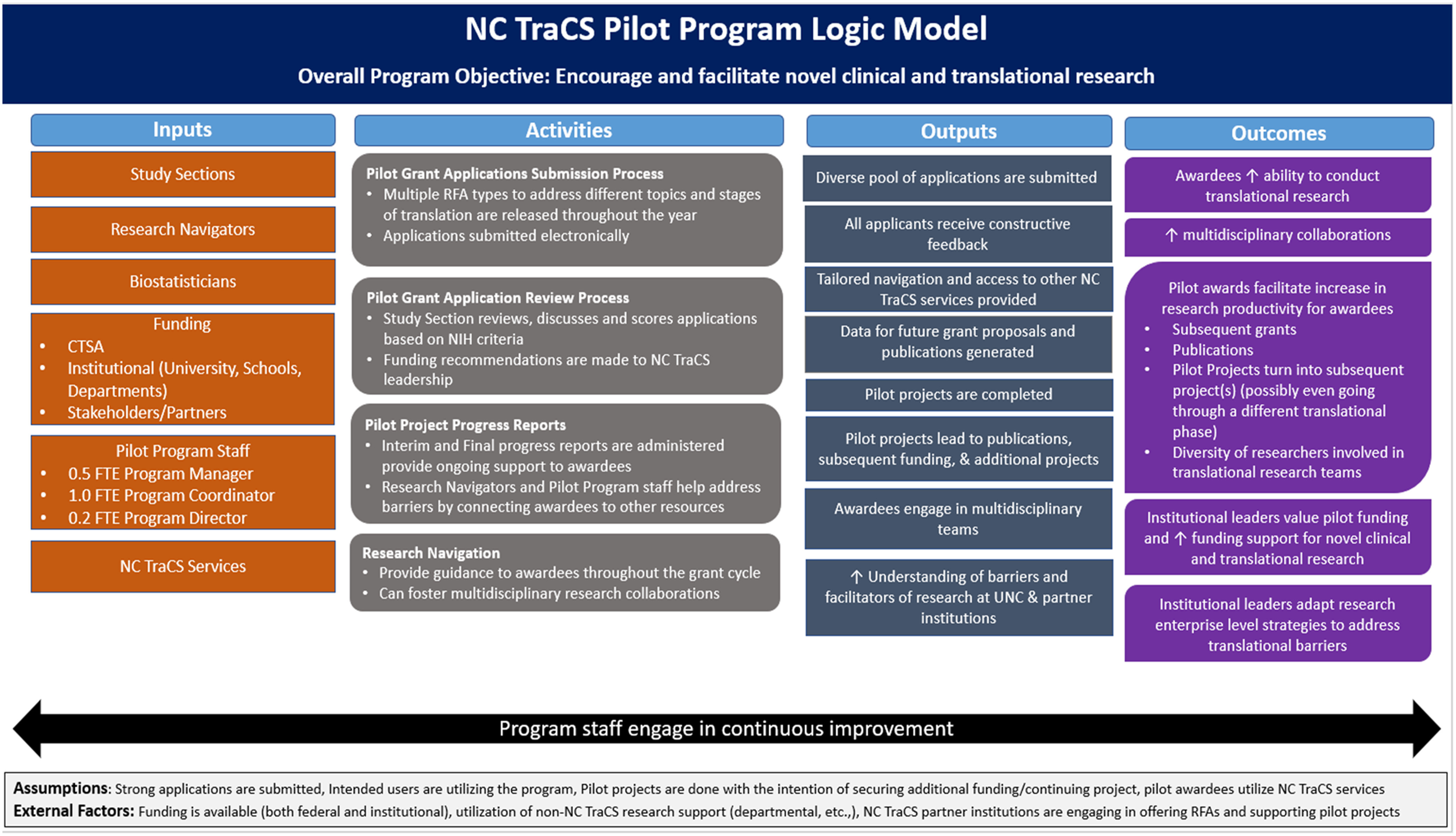
NC TraCS Pilot Program logic model.

The NC TraCS Pilot Program is open to UNC-CH and partner institution researchers that are addressing the development of therapies, diagnostics, or devices applicable to human disease, clinical research/trials, epidemiological studies, and/or community-based research. The NC TraCS Pilot Program allows early stage investigators (trainees and junior faculty) or established investigators who are trying new research directions and/or forming a new multidisciplinary team to access research funding. The overall goals of these pilot grants are to allow investigators to generate preliminary data, evaluate how the data will be analyzed and validated, and clarify human and financial resources needed to conduct a more comprehensive research project [5–8]. Therefore, it is expected that these pilot grants will result in publications and extramural funding to continue the clinical and translational research. A complete logical framework illustrating key program components and expected outcomes is presented in Fig. 1.

Regardless of how the individual pilot programs are administered across the CTSA consortium, CTSA pilot programs need to be able to solicit strong applications, select high-quality projects, and track outcomes associated with the pilot funding. However, there is limited information and data related to the structure and impact of pilot programs administered by the CTSA hubs [9,10]. The case study presented here illustrates how we have operationalized both our NC TraCS Pilot Program and evaluation of that program.

### How is the NC TraCS Pilot Program Operationalized?

The NC TraCS Pilot Program provides three major types of pilot grants: small $2,000 ($2K), $5,000–$50,000 Translational Research Matched Pilot Grants ($5K–$50K), and Specialized Pilot Grants. The $2K pilots are awarded monthly to UNC-CH faculty, doctoral students, postdoctoral trainees including clinical fellows, and faculty of NC TraCS and partner institutes to assist with implementing a proposed study or moving a research project forward. The $2K pilot awards provide rapid access to funds to support promising and innovative translational research. The $5K–$50K grants are awarded three times a year and generally require matching funds from the applicant’s home department, center, school, or another partnering institution. The $5K–$50K grants are aimed at encouraging and facilitating novel clinical and translational research and are provided to UNC-CH faculty (members of NC TraCS partner institutes and community organizations can be collaborators). The $2K and $5K–$50K grants are not research topic or institution-specific, although priority is given to proposals that focus on state health priorities such as maternal and infant health, substance abuse, oral health, chronic disease, in accordance with the NC TraCS mission to improve the health of North Carolinians. The Specialized Pilots, on the contrary, are released as necessary to fund research projects focused on one of the CTSA or UNC-CH strategic initiatives and are available for UNC-CH faculty (members of NC TraCS partner institutes and community organizations can be collaborators). Upon request, multi-CTSA specialized request for applications (RFAs) are also released as Specialized Pilots. The Specialized Pilots occur at various frequencies, with some being one-time offerings, depending on the emerging need. Regardless of the grant type, most grants are for 1 year, with requests for no-cost extensions reviewed on a case-by-case basis by the Pilot Program staff. The frequency, aim, and requirements of all pilot grant types can be found in Supplemental Table 1.

Potential applicants are encouraged to utilize NC TraCS research support services, particularly Research Navigators, Biostatistics, and Proposal Development, while preparing their applications. Research Navigators are available to everyone seeking guidance on research support services available and/or consultation on moving their research idea forward. Research Navigators can connect the investigators to other NC TraCS and institutional resources that might assist in supporting their research needs. Biostatistical consultations can be used by pilot grant applicants to assist with methodology and data analysis plans presented in the grant applications. If the investigator is resubmitting an application, they also seek biostatistician support to address any concerns raised during the initial review. The Proposal Development service can assist with all aspects of the grant writing process and is utilized by pilot grant applicants to review proposals for content as well as language/style. Members of the Proposal Development team can review applications and advise on study aims, study design, and consistency with RFA guidelines and priorities.

**Fig. 2.**
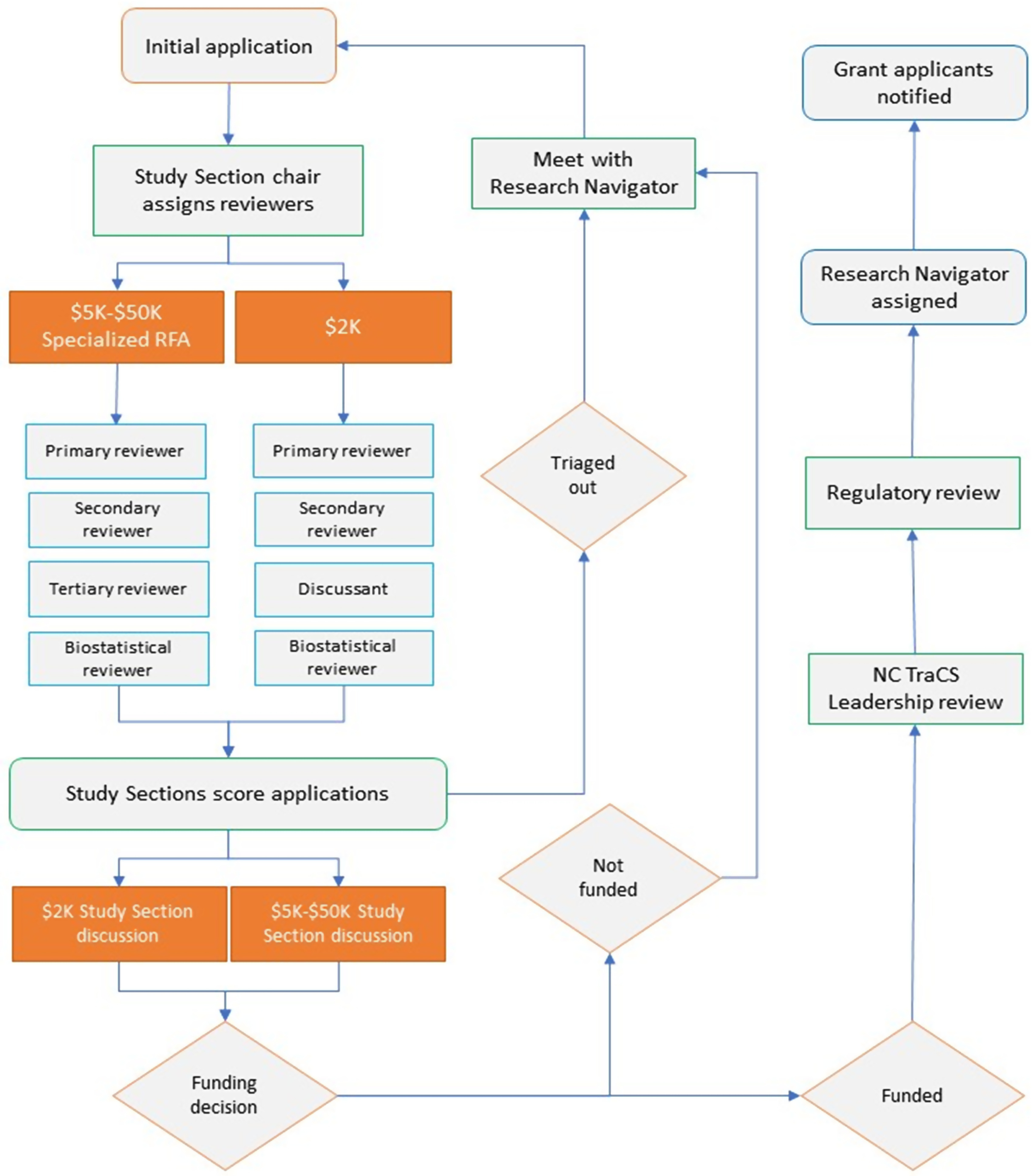
Pilot program application process.

Upon submission, each of the NC TraCS Pilot Program applications is subject to a rigorous review by either the $2K or $5K–$50K Study Sections, as shown in Fig. 2. The Specialized Pilot applications follow the same procedure as the $5K–$50K applications. The $2K Study Section meets monthly and consists of six members, including faculty members, a biostatistician, an expert from a partner institution, NC TraCS Research Navigators, and NC TraCS service leaders. For the $2K proposals, scores from all four reviewers (primary, secondary, tertiary, and biostatistician) are averaged together. The $5K–$50K Study Section meets every 4 months and consists of approximately 25 senior faculty members from UNC-CH and partner institutions with diverse expertise, backgrounds, and experience with participating in federal study sections. For $5K–$50K applications discussants, primary and secondary reviewers are assigned according to subject matter expertise, with consideration for the number of applications per reviewer. All $5K–$50K applications are also scored and reviewed by a biostatistician.

Once preliminary scores from reviewers (for all grant types) have been entered into a cloud-based grant management program, the Study Section completes their review of the proposals, after which the Study Section chairs triage out the bottom 40–50% of applications as not moving forward. The remaining applications are discussed and scored by all Study Section members using an NIH-based 1–9 scoring scale, along with any additional pilot award-specific criteria (described in Supplemental Table 1). The Study Section co-chairs review the final scores and recommend applications for funding to NC TraCS leadership. At the end of each review, a detailed written critique is provided to all applicants, regardless of being triaged out.

Lead Principal Investigators (PIs) of each of the selected applications are notified about their application funding status, assigned a Research Navigator, and receive Study Section scientific summaries and Regulatory review summaries to address any concerns. For all funded pilot projects, Institutional Review Board (IRB) and Institutional Animal Care and Use Committee (IACUC) reviews are conducted by the NC TraCS Regulatory service to identify potential issues. However, although NC TraCS Regulatory service is available to assist, regulatory submissions and approvals are the responsibility of funded PIs. Upon IRB and/or IACUC approval and NIH clearance, the pilot project can start the project activities.

Low scoring but promising applications are encouraged to consider resubmitting and are invited to a consultation with a Research Navigator and specific services as recommended (identified during the review) at NC TraCS. Applicants for all grant types may re-submit their unsuccessful grant application to NC TraCS one time and are strongly encouraged to consult with an NC TraCS Research Navigator prior to resubmission. Resubmissions are reviewed by the original reviewers when possible, and responsiveness to the original review comments is a major criterion during the resubmission review.

### Guiding Evaluation Theory and Framework

The aim of the NC TraCS Pilot Program is “to encourage and facilitate novel clinical and translational research in its many forms.” Evaluation of the NC TraCS Pilot Program is focused on assessing the effectiveness of the program and the impact it has on research productivity. The key evaluation questions, therefore, are: (1) What types of pilot grants are offered?; (2) who and to what extent is utilizing the pilot program?; (3) what are the contributing factors for a successful pilot project?; and (4) what are the outcomes of the pilot projects? We set out to answer these questions by aligning them with the intended outputs and outcomes of the program presented in the logical framework (Fig. 1), using a comprehensive list of evaluation measures found in Table 1.

**Table 1.** NC TraCS Pilot Program evaluation measures

Type of measure	Evaluation questions?	Measure	Measure definition
Process	What types of pilot grants are offered?	Type of pilot RFAs released	Type and counts of pilot RFAs released
	Who and to what extent is utilizing the pilot program?	Application volume	Number of applications submitted (initial and resubmission) by RFA type
		Applicant and Awardee demographics	Demographics such as Investigator level (junior, middle, senior), Primary role, Institutional affiliation, School affiliation, and Departmental affiliation for lead PI applicants.
		Funding rate	Three different funding rates are calculated for each RFA type and overall pilot program. • Initial = initially funded/initially submitted • Resubmission = resubmissions funded/resubmissions submitted • Overall = total funded (initial + resubmission)/initial submissions
	What are contributing factors for a successful pilot project?	NC TraCS service usage	Type and counts of services used by pilot applicants in preparation for pilot application. Type and counts of services used by pilot awardees after being funded.
		Barriers and Facilitators to Translational Research	Quantitative and qualitative data from awardees collected at the 6th month and end of the pilot grant cycle.
Outcome	What are the outcomes of the pilot projects?	Percentage of pilots awarded reporting completion of the project	Percentage of NC TraCS-funded pilot projects that report having completed the pilot project at the close-out survey.
		Publications related to pilot project	List of publications for which pilot project played a contributing role, according to the NC TraCS-funded pilot awardee.
		Subsequent funding amount related to pilot project	List of subsequent funding awards and amount of funding received for which NC TraCS-funded pilot project played a contributing role, reported by pilot awardees and confirmed via NIH Reporter.
		Percentage of pilots with at least one publication	Percentage of NC TraCS-funded pilot projects that have been linked to at least one publication.
		Percentage of pilots with at least one subsequent funding award	Percentage of NC TraCS-funded pilot projects that have led to at least one subsequent funding award.
		Return on investment	Measure calculated using the following formula: Amount of subsequent funding linked to NC TraCS-funded pilots/Amount of funding provided through NC TraCS Pilot Program (direct + match funding)

RFA, request for application.

We utilized a developmental approach to implement our proposed evaluation plan. Developmental evaluation approach places evaluators on the program team as collaborators who facilitate systematic data-based reflection and decision making throughout the cycle of the program. [11,12] We chose this evaluation approach because it allows us to focus on both process and outcome evaluations in a way that allows for real-time sharing with the program leadership so that they refine their strategy on an ongoing basis and adapt new pilot RFAs accordingly.

## Evaluation Study of NC TraCS Pilot Program

### Data Measures

The evaluation measures presented in Table 1 formed the foundation for this analysis. Number and type of pilot RFAs released between 2009 and 2019 were categorized as $2K, $50K, or Specialized RFAs. Total volume of applications is represented by the number of initial application submissions. Initial applications were defined as first-time grant applications. Resubmission applications were defined as first-time grant applications that were initially not funded and subsequently resubmitted during a later RFA round. Application data including demographic information (PI name, PI affiliation, position title, etc.) for lead PIs, funding status, and funding amount for all grants administered between 2009 and 2019 were abstracted from the pilot grants tracking system. Lead PIs position title, grant type, and application status were used as primary descriptive measures. Total grant funding per year was broken down into funds that NC TraCS administered and matching funds received from other sources, when applicable. NC TraCS service utilization (previous and planned), pilot project completion, and barriers/facilitators to research were self-reported by awardees from 2015 to 2019 as part of their final progress reports. Number and percentage of pilots with at least one linked publication was calculated as part of the Common Metrics Initiative and is reported as a cumulative measure up to 2018. Number of pilots with at least one subsequent funding and the amount of subsequent funding was self-reported by awardees and verified by Pilot Program staff using NIH Reporter.

### Data Analysis

Funding rates were calculated for initial applications and resubmitted applications and were stratified by PI position title and grant type using STATA^®^ and MS Excel^®^. Overall funding rate was calculated using the total number of funded applications (initial + resubmissions), which was then divided by the total number of initial applications. Application frequency by position title was calculated by dividing the number of applications for a particular grant type by the total number of applications stratified by position title. Graduate students, postdoctoral trainees, residents, fellows, and undergraduate students (who are eligible for the $2K awards) were combined into a single trainee category. The “Other” position title category contained applicants that either did not have a position title listed or did not fit into any other categories.

Qualtrics was used to administer final progress reports at the end of each pilot award. Lead PIs were asked to complete the questionnaire and self-report on NC TraCS service utilization, completion status of the project, barriers/facilitators of research, publications linked to the pilot project, and subsequent funding received or submitted. NC TraCS pilot program staff followed up with Lead PIs that reported the submission of publications and subsequent funding to monitor the status of those products. Additionally, NC TraCS service utilization for resubmitted applications was abstracted from the pilot grants tracking system where applicants self-reported service utility.

Number and percentage of pilots with at least one linked publication was calculated as part of the Common Metrics Initiative and is reported as a cumulative measure up to 2018. Estimated return on investment was calculated for 2009–2019 using the following formula: Amount of subsequent funding linked to NC TraCS-funded pilots/Amount of funding provided through NC TraCS Pilot Program (direct + match funding). MS Excel^®^ was used to conduct a descriptive analysis for these measures.

### Data Limitation

As the NC TraCS Pilot Program encourages multidisciplinary teams, pilot applications may have multiple PIs and co-investigators. For the purpose of this study, we focused our analysis on the individual listed as the lead applicant and the demographics associated with that individual. Another limitation to our data is historical shifts in eligibility criteria for our pilot RFAs. Early on in the NC TraCS Pilot Program, trainees were eligible to apply for all grant types, however, several years into the program, eligibility requirements changed, and trainees were only eligible to submit $2K grants as lead PIs. An exception to these eligibility criteria was made if the trainee was being considered for a UNC-CH faculty position at the time of application. At the time of this analysis, data on NC TraCS service utilization was only available for resubmissions through the grant applications process, therefore we are only able to present pre-award utilization data for grants that were resubmitted.

## Summary of Findings

### Applications

From 2009 to 2019, the NC TraCS Pilot Program received a total of 2,343 unique applications, of which 440 were resubmitted (Table 2). In total, 2,783 applications were scored by reviewers from 2009 to 2019. The $5K–$50K grants made up 40.2% (*n* = 943) of all grant applications, followed by the $2K at 39.6% (*n* = 928) and Specialized Pilot RFAs at 20.1% (*n* = 472). As seen in Table 2, assistant professors submitted the highest number (*n* = 813) of applications, followed by trainees (*n* = 608), associate professors (*n* = 434), and professors (*n* = 383). For $5–50K pilots, the highest number of applications were submitted by assistant professors (*n* = 408), followed by associate professors (*n* = 201) and professors (*n* = 180). Similarly, for the $2K grants trainees submitted the largest volume of applications (*n* = 504) followed by assistant professors (*n* = 231), associate professors (*n* = 95), and professors (*n* = 64). Assistant professors (*n* = 174) also responded with the largest volume for Specialized Pilot applications followed by professors (*n* = 139) and associate professors (*n* = 138). Overall, out of the 2,343 distinct applications, the program awarded 933 grants, 276 of which were funded after the application was resubmitted.

**Table 2.** Pilot grant funding rates by position title and grant type

	Position	Trainee *n* = 608 (funded/submitted)	Research staff *n* = 64 funded/submitted)	Instructor = 28 (funded/submitted)	Assistant professor *n* = 813 (funded/submitted)	Associate professor *n* = 434 (funded/submitted)	Professor *n* = 282 (funded/submitted)	Other *n* = 13 (funded/submitted)	Total sample *n* = 2343 (funded/submitted)
Total	Initial proposals (*n* = 2343) *(initially funded/initially submitted)*	28.8% (175/608)	26.6% (17/64)	25% (7/28)	25.8% (210/813)	26.7% (116/434)	33.9% (130/383)	15.4% (2/13)	28% (657/2343)
	Resubmission proposals (*n* = 440) *(resubmissions funded/resubmissions submitted)*	66.2% (90/136)	52.2% (12/23)	0% (0/3)	68.2% (105/154)	56.2% (41/73)	54.9% (28/51)	0% (0/0)	62.7% (276/440)
	Overall *(total funded/initially submitted)*	43.6% (265/608)	45.3% (29/64)	25% (7/28)	38.7% (315/813)	36.2% (157/434)	41.3% (158/383)	15.4% (2/13)	39.8% (933/2343)
2K	Initial proposals *n* = 928	30% (151/504)	42.9% (6/14)	27.3% (3/11)	27.7% (64/231)	37.9% (36/95)	39.1% (25/64)	22.2% (2/9)	30.9% (287/928)
	Resubmitted proposals *n* = 171	71.2% (84/118)	33.3% (1/3)	0% (0/0)	75% (27/36)	70% (7/10)	75% (3/4)	0% l(0/0)	71.3% (122/171)
	Overall	46.6% (235/504)	50% (7/14)	27.3% (3/11)	39.4% (91/231)	45.3% (43/95)	43.8% (28/64)	22.2% (2/9)	44.1% (409/928)
$5K–$50K	Initial proposals *n* = 943	23.7% (23/97)	14.6% (6/41)	15.4% (2/13)	24% (98/408)	23.9% (48/201)	29.4% (53/180)	0% (0/3)	24.4% (230/943)
	Resubmitted proposals *n* = 232	33.3% (6/18)	55% (11/20)	0% (0/3)	70.5% (74/105)	54.2% (26/48)	57.9% (22/38)	0% (0/0)	59.9% (139/232)
	Overall	29.9% (29/97)	41.5% (17/41)	15.4% (2/13)	42.2% (172/408)	36.8% (74/201)	41.7% (75/180)	0% (0/3)	39.1% (369/943)
Special RFAs	Initial proposals *n* = 472	14.3% (1/7)	55.6% (5/9)	50% (2/4)	27.6% (48/174)	23.2% (32/138)	37.4% (52/139)	0% (0/1)	29.7% (140/472)
	Resubmitted proposals *n* = 37	0% (0/0)	0% (0/0)	0% (0/0)	30.8% (4/13)	53.3% (8/15)	33.3% (3/9)	0% (0/0)	40.5% (15/37)
	Overall	14.3% (1/7)	55.6% (5/9)	50% (2/4)	29.3% (52/174)	28.3% (40/138)	39.6% (55/139)	0% (0)	32.8% (155/472)

RFA, request for application.

### Funding Rates

The overall funding rate for all pilot applications was 39.8% (657 initially funded + 276 resubmissions funded/2343 initially submitted applications) compared to 28% (657 initially funded/2343 initially submitted applications) funding rate for initial submission and 62.7% (276 resubmitted applications funded/440 applications resubmitted) for resubmissions. As illustrated in Table 2, professors (33.9%) had the highest initial funding rate, followed by trainees (28.8%), associate professors (26.7%), and assistant professors (25.8%). As per funding rate by pilot type, $2K pilot grants had the highest initial (30.9%), resubmission (71.3%), and overall (44.1%) funding rates.

### Resubmissions

One-thousand six-hundred and eighty-six initially submitted pilot grant applications were not funded, and 440 of those applications were revised and subsequently resubmitted. Among the 440 resubmissions, assistant professors (*n* = 154) submitted the largest volume, followed by trainees (*n* = 136), associate professors (*n* = 73), and professors (*n* = 51). Trainees (*n* = 118) submitted the largest volume of resubmissions for $2K grants, followed by assistant professors (*n* = 36) and associate professors (*n* = 10). For $5K–$50K resubmissions, assistant professors (*n* = 105) had the largest volume of resubmissions, followed by associate professors (*n* = 48) and professors (*n* = 38). Resubmission volumes were generally proportional to initial submission volumes when stratified by position title. For resubmission funding rates, assistant professors (68.2%) had the highest rate followed by trainees (66.2%), associate professors (56.2%), and professors (54.9%).

### NC TraCS Service Utilization

Between October 2015 and August 2019, out of the 223 awardees who completed final progress reports, 65.5% of pilot awardees (*n* = 220) used NC TraCS services during the course of their pilot grant. 62.4% of the same group indicated that they were planning on using NC TraCS services for their projects in the future. Of all NC TraCS services used during the course of pilot grants, Proposal Development (*n* = 26), Biostatistics (*n* = 25), Biomedical Informatics (*n* = 18), Research Navigators (*n* = 16), and Recruitment and Retention (*n* = 14) were the most commonly used. When asked about services that they planned to use in the future awardees selected Proposal Development (*n* = 27), Biostatistics (*n* = 26), Biomedical Informatics (*n* = 23), Core Laboratory Facilities and Translational Technologies (*n* = 14), and Research Navigators (*n* = 10).

**Fig. 3.**
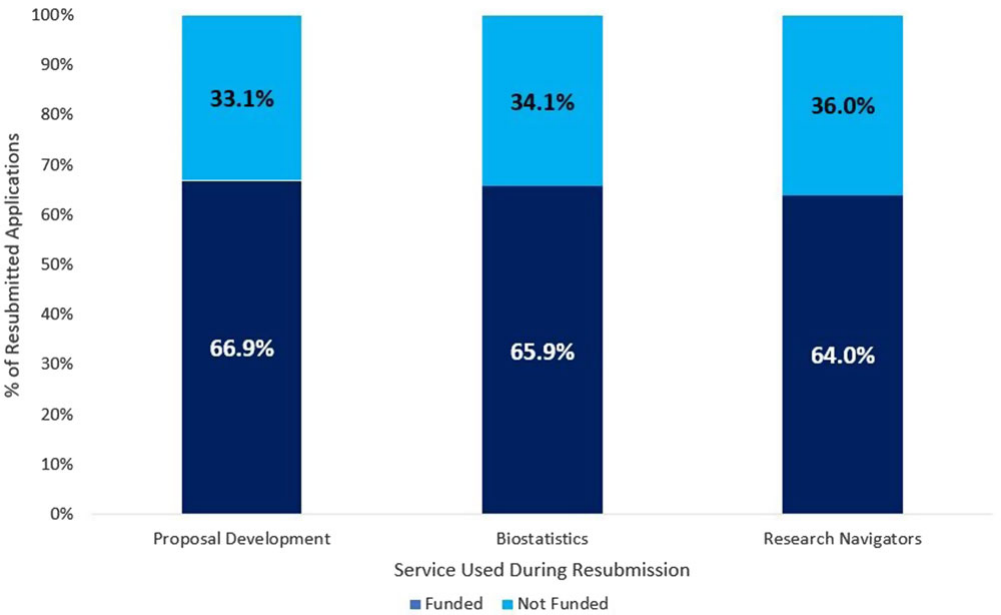
Resubmission funding status by service utilization.

Resubmission applicants are asked to report on their utilization  of NC TraCS services in preparation for their pilot application. Between 2009 and 2019, a total of 269, or 61.1% of resubmission applicants utilized NC TraCS services when preparing their applications for resubmission. As shown in Fig. 3, Proposal Development (*n* = 148), Biostatistics (*n* = 126), and Research Navigators (*n* = 114) were the three most commonly utilized NC TraCS services for applicants resubmitting pilot grant proposals. Resubmissions that utilized Proposal Development, Biostatistics, or Research Navigators were funded approximately 65% of the time.

### Barriers and Facilitators to Research

From 2015 to 2019, awardees reported recruitment issues, delays with regulatory processes, and staffing/personnel issues as some of the most common roadblocks during the course of their pilot project. Awardees also reported that NC TraCS Research Navigators, biostatisticians, biomedical informatics analysts, regulatory specialists, and community engagement specialists were instrumental in not only successfully completing their pilot projects, but also in preparing for subsequent grant proposals, publications, and commercialization efforts.

### Funding Amounts

**Fig. 4.**
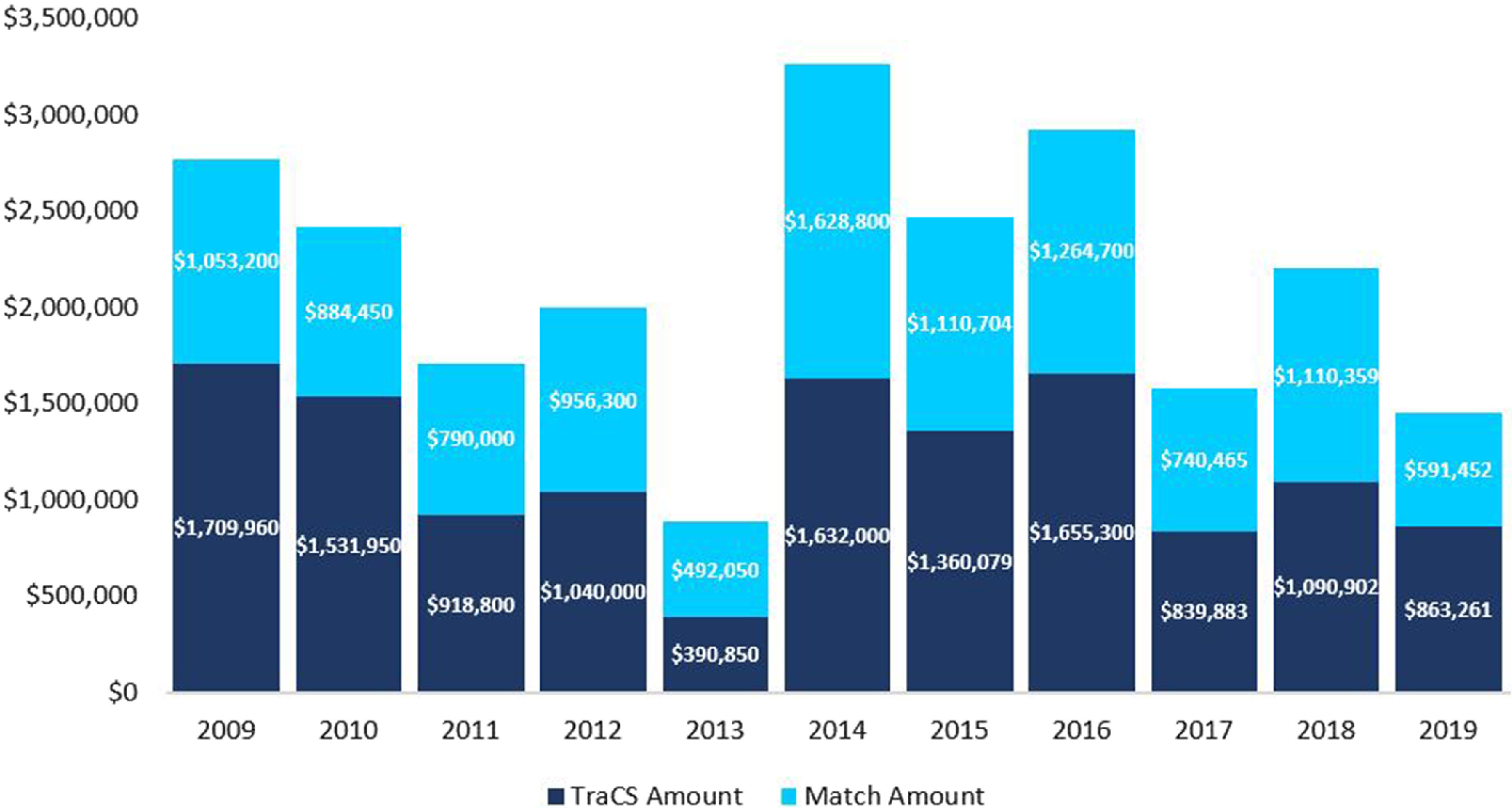
Pilot grant funding awarded per year.

Although the amount of funding awards has varied over years, a total of $23,655,464 has been awarded to the 933 NC TraCS pilots that were successfully funded between 2009 and 2019 (Fig. 4). The amount directly administered by NC TraCS Pilot Program is $13,032,986, the rest is matching funding from UNC-CH departments, partner institutes, community/industry partners, and other CTSA hubs.

### Research Productivity

Out of the 223 awardees who completed final progress reports between 2015 and 2019, 70% reported completing their pilot projects as outlined in the application. As of 2018, 25% of awarded pilots had resulted in at least one publication. The $23.4 million that was invested in direct grants and matching funds into 933 projects, has generated data instrumental in obtaining $219 million in new grant funding, yielding a return on investment of $9.36 for every $1 spent.

## Discussion

Utilizing a developmental evaluation approach and a framework based on programmatic design, this process evaluation has been useful for NC TraCS Pilot Program to gain a better understanding of how the program is functioning and whether or not it is on track to fulfilling its mission, while contributing to the overall mission of NC TraCS and CTSAs. Although more research is needed, we found that the combination of offering a variety of pilot funding opportunities, a rigorous review process, and access to Biostatistics, Proposal Development, and Research Navigator services are key factors in the overall success of our pilot grant program.

### Successful Program Operationalization

Since pilot program RFAs and application processes are the main program activity that the largest number of researchers interact with, we were interested in understanding if offering a wide diversity of RFAs contributed to successful operationalization. From 2009 to 2019, the program received 2,343 unique applications and ultimately funded 933 projects. Overall, assistant professors submitted the largest volume of applications, which indicates the existence of gaps and lags in funding that have been found to exist for junior faculty [13]. It is also promising to see that over half of the applications for the $2K grants were from trainees, as this represents a large group of individuals that are getting exposure to CTSA resources early in their development as researchers. Application volume data stratified by position title indicates that the NC TraCS Pilot Program is meeting funding gaps differently for each of its users through the wide range of pilot grant types it offers. The specialized RFAs allow investigators to apply for more targeted funding streams and were utilized primarily by senior faculty. The NC TraCS Pilot Program also allows graduate students and nonfaculty researchers to apply for $2K grants, which exposes junior and developing researchers to both an NIH-style grant application review and CTSA services and support. It is noted that the majority of the applicants for the more generalized $2K and $5K–$50K grants were assistant professors and trainees. On the contrary, professors were more likely to apply for specialized grants, which were offered to address specific topics, cultivate cross-institutional collaborations, and highlight innovative centers and research across UNC-CH.

This analysis found an initial funding rate of 28% among pilot applications submitted between 2009 and 2019, while the funding rate among resubmissions was twice as high. Furthermore, the resubmission funding rates for assistant professors (68.6%) were actually higher than those of associate professors (56.2%) and professors (54.9%). In contrast, professors had the highest initial funding rate (33.9%) compared to assistant professors (25.8%) and associate professors (26.7%). Upon reviewing this data with the program leadership, it became evident that the operationalization of our pilot program plays a vital role in explaining why we are seeing these differences. There are at least three factors that can contribute to this difference between initial and resubmission funding rates: review process, education, and resources provided to investigators during the application process. While these factors positively impact all levels of investigators, they are designed to be particularly impactful for early stage investigators in accordance with the NC TraCS Pilot Program’s aim of supporting early stage investigators. Further analysis of reviewer scores might provide additional insight on both the quality of these applications and the role the rigorous NIH-style review process plays in selecting strong applications that are initially funded.

We did find that the utilization of key NC TraCS services during the resubmission process contributed to the marked increase in the resubmission funding rate compared to the initial funding rate. Trainees and assistant professors both utilized Biostatistics, Proposal Development, and Research Navigator services more than associate professors and professors, illustrating areas where there are gaps in early stage investigator knowledge and skills that NC TraCS can help bridge. The funding rate for resubmitted applications was 62.7%, with 61.1% of resubmitted applications receiving assistance from additional NC TraCS services prior to resubmission.

A Research Navigator is assigned to each funded pilot to support the PIs through the pilot administration process. The Research Navigator stays in contact with the funded pilot PIs to track progress and help mediate any challenges that might arise by connecting the PIs to appropriate resources. This tracking occurs through three formal meetings but also on an ad hoc basis, as needed. Furthermore, the funded pilot PIs are also asked to complete a mid-point (interim) and final progress report to provide feedback on progress, roadblocks encountered, with a focus on those that NC TraCS staff can assist with, and report any planned or submitted extramural grants and publications. From 2015 to 2019, among those who responded to their final progress report, 70% reported that they had successfully completed their projects as outlined in the application. The high pilot project completion rate indicates that NC TraCS Research Navigators are an important aspect of how our pilot program is operationalized, although further evaluation and analysis are needed to determine the exact mechanisms and impacts that these components have on overall project success.

Support via NC TraCS services during the pilot grant cycle was also an important and intentionally designed component of the program. Between 2015 and 2019, 65.5% of awardees used NC TraCS services while conducting their pilot projects. Additionally, a majority of awardees reported that they were planning to continue using NC TraCS services for their project after the conclusion of the pilot grant cycle. The ability to leverage and connect pilot awardees to additional NC TraCS services is an important aspect of this program, and positively contributes to the successful completion of pilot projects.

### Pilot Program Impact on Research Productivity

While evaluating outcome measures was not the main focus of this analysis, preliminary metrics indicate that funding pilots are a good investment with an estimated return on investment of $9.36 for every $1 spent. Data on subsequent funding linked to pilot projects are also reported through final progress reports and then manually monitored through institutional funding data sources. An estimated return on investment is calculated based on the limited data available. Similarly, publications that are linked to NC TraCS-funded pilots are reported through final progress reports and manual checks of institutional news outlets. Using the estimated information that is available, percentage of pilot projects with at least one publication is reported to the Common Metrics Initiative of NCATS. However, a more robust data collection and analysis approach is needed to truly understand the impact our pilots are having on research productivity, both for the research projects and the researchers themselves. The next phase of our evaluation study will prioritize establishing a systematic approach to collecting and reporting on these outcome measures.

## Conclusion

The NIH has previously estimated that there is a 4–7-year lag between taking an academic position and receiving an extramural grant, and our findings suggest that this is generally true for junior faculty members at UNC-CH [13]. Pilot funding aids early stage investigators in the development of their research and decreases the amount of time between appointments and receiving extramural funding [13–15]. The NC TraCS Pilot Program is designed to both serve investigators and address gaps in translational research, while fulfilling the traditional pilot grant program’s intention of providing funding to generate preliminary data, plan for data analysis, validate methods, and estimate required financial and human resources for future work. This case study illustrates the importance of creating an evaluation framework according to program design while also utilizing both process and outcome measures to quantify success.

## Supplementary material

For supplementary material accompanying this paper visit https://doi.org/10.1017/cts.2020.557. click here to view supplementary material
