# Supplementary material for: Evaluating a CTSA-funded pilot grant program
Source: J Clin Transl Sci. 2020 Nov 16;5(1):e63. doi: 10.1017/cts.2020.557 (PMC8057500; doi:10.1017/cts.2020.557)
Supplement: Supplementary file 1 [file S2059866120005579sup001.docx]

**SUPPLEMENTARY MATERIAL**

Supplemental Table 1: NC TraCS Pilot Grant Types

|  | **$2K** | **$5K-$50K** | **Specialized Pilot RFA** |
| --- | --- | --- | --- |
| **Frequency** | Monthly | 3x/year | Varies |
| **Pre-Application Requirement** | None | None | Varies |
| **Matching Funds** | Not Required | Required | Required* |
| **Grant Purpose** | To assist in the implementation of a study or move an existing study forward, and to assist with intensive consultations services from health research stakeholders. | To provide stimulus for new research initiatives to obtain preliminary data for new applications for extramural funding. | To further innovative technologies to market, facilitate novel research and collaborations with other institutions, to stimulate investigators to serve as PIs for new centers, facilitate the creation of inter-disciplinary teams. |
| **Grants Included** | $2K; Stakeholder Engagement RFAs | $50K (2009-2011); $10K (2009-2011); $5K-$50K (2011-present) | NC TraCS medical technology development RFAs, center-based RFAs, Collaborative RFAs for partner institutions |
| **Review Criteria** | -Significance  -Novelty/innovation  -Relevance to translational research  -Potential for future grants, publications and/or commercialization  -Soundness of methods  -New direction if senior PI  -Feasibility of accomplishing goals within project period | -Same as above, plus:  -Level of community engagement (if applicable)  -Utilization of NC TraCS services and resources (if applicable)  -Focus on one of the NC health priorities | Varies depending on RFA |
| **Who can apply?** | Trainees, Faculty, Clinicians, Collaborators from partner institutions** | Faculty, Clinicians, Collaborators from partner institutions** | Faculty, Clinicians, Collaborators from partner institutions** |

*For the majority of Specialized Pilot RFAs

**Collaborators from partner institutions can serve as PIs on pilot grants, but applications must include UNC-CH investigators as collaborators
